# A robust machine learning approach to predicting remission and stratifying risk in rheumatoid arthritis patients treated with bDMARDs

**DOI:** 10.1038/s41598-025-09975-z

**Published:** 2025-07-04

**Authors:** Fatemeh Salehi, Emmanuelle Salin, Benjamin Smarr, Sara Bayat, Arnd Kleyer, Georg Schett, Ruth Fritsch-Stork, Bjoern M. Eskofier

**Affiliations:** 1 Machine Learning and Data Analytics Lab, Department Artificial Intelligence in Biomedical Engineering, Friedrich-Alexander-Universität Erlangen-Nürnberg, 91052 Erlangen, Germany; 2 Halıcıoğlu Institute for Data Science, La Jolla, CA USA; 3 Department of Internal Medicine 3, Rheumatology and Immunology, Universitätsklinikum Erlangen, 91054 Erlangen, Germany; 4 Department of Rheumatology and Clinical Immunology, Charité – University Medicine Berlin, 10117 Berlin, Germany; 5 BIOREG, Health Care Centre Mariahilf, ÖGK and Rheumatology Department, Sigmund Freud Private University, 1060 Vienna, Austria

**Keywords:** Rheumatology, Bioinformatics, Biomedical engineering

## Abstract

Rheumatoid arthritis (RA) is a chronic autoimmune disease affecting millions worldwide, leading to inflammation, joint damage, and reduced quality of life. Although biological disease-modifying antirheumatic drugs (bDMARDs) are effective, they are costly, and up to 40% of patients do not achieve remission within six months. Accurate prediction of treatment response is crucial for optimizing care, minimizing side effects, and enhancing cost efficiency. This study proposes a robust machine learning framework for predicting six-month remission in RA patients using baseline routine clinical data. The framework also integrates risk stratification and explainability to enhance its clinical applicability. We evaluated multiple machine learning models, AdaBoost, Random Forest, XGBoost, and Support Vector Machines, using data from Austrian RA patients. We externally validated the results on an independent dataset from the Erlangen Hospital. To improve the reliability of probability estimates for actionable risk stratification, we employed calibration techniques, including Platt scaling, Isotonic regression, Beta calibration, and Spline calibration. We generated calibration curves to assess and visualize the alignment between predicted probabilities and observed outcomes. In addition, we used SHapley Additive exPlanations (SHAP) to analyze the contributions of different patient characteristics to the prediction of RA remission. AdaBoost demonstrated stronger performance than the other models, achieving an accuracy of 85.71% and a Brier score of 0.13 with isotonic regression calibration. SHAP identified DAS28, visual analog scales (VAS), age, and swollen joint count (SJC) as important characteristics for the prediction of RA remission. We also stratified patients into low-, medium-, and high-risk categories based on model predictions to support follow-up scheduling and treatment prioritization. Our framework predicts RA remission before the initiation of bDMARD therapy. It enables personalized care, actionable risk stratification, and optimized resource allocation. Its robustness was validated on two different individual cohort datasets, which highlights its potential for integration into routine clinical workflows.

## Introduction

Rheumatoid arthritis (RA) is a chronic inflammatory condition that affects 40 to 80 million people worldwide. It primarily impacts the small joints of the hands and feet, causing pain, stiffness, and reduced mobility. Beyond joint damage, RA can exhibit systemic features, causing significant physical disability and a diminished quality of life^1–3^.

Biological disease-modifying antirheumatic drugs (bDMARDs) are highly effective in treating RA, particularly for patients in whom conventional synthetic DMARDs (csDMARDs) fail to provide adequate disease control. During bDMARD therapy, patients are typically monitored every 1 to 3 months, with treatment adjustments recommended according to EULAR guidelines if remission is not achieved within six months^4^. Despite the efficacy of bDMARDs, up to 40% of RA patients fail to achieve remission within six months^4,5^. For these non-responders, disease progression leads to increased healthcare costs and reduced quality of life^6–9^. Although bDMARD costs have decreased, they remain expensive, emphasizing the need for targeted use^10^. Early prediction of treatment response is essential to improving patient outcomes, minimizing side effects, and enhancing cost-efficiency^11–14^.

Accurate prediction of remission before treatment initiation is a critical component of personalized medicine. By tailoring therapies to individual patients, clinicians can avoid prolonged trial-and-error periods, unnecessary medication exposure, and potential side effects. This approach helps optimize outcomes and resource allocation. Recently, machine learning has emerged as a valuable tool in rheumatology, identifying patterns and predicting treatment responses in RA patients^15,16^.

However, several challenges hinder the implementation of RA remission prediction models in clinical practice. Many models rely on non-routine clinical data, such as gene expression or imaging data^17–20^. While these datasets enhance accuracy, their high cost and limited accessibility reduce their practical utility in real-world clinical settings. Timing is another challenge. EULAR guidelines recommend treatment adjustments if remission is not achieved within six months of bDMARD initiation^4^. However, many studies assess remission at 12 months or later, which diminishes their clinical relevance^21^. While existing models predicting six-month remission using routine clinical data are insightful, many rely on small sample sizes^13,22^, which limits their generalizability. Larger and more diverse datasets are needed to enhance the robustness of these models and their ability to generalize across a wider range of clinical scenarios. Furthermore, to our knowledge, no studies have externally validated RA remission prediction models using independent datasets from diverse populations. External validation is essential to evaluate model performance on unseen data and ensure its generalizability across different clinical settings. Without this step, the utility of such models remains limited. Beyond binary predictions of remission, clinicians would benefit from insights into the probability of remission and the stratification of patients based on their risk. These probabilities could guide personalized follow-up schedules and treatment plans. However, most existing models are limited to binary remission predictions and lack risk stratification capabilities.

In this study, we present a machine learning framework designed to predict RA treatment outcomes while addressing the key limitations of existing methods. This framework utilizes independent datasets for external validation, incorporates risk stratification based on calibrated probability estimates, and provides feature importance analyses to support informed clinical decision-making.

## Methods

### Study population and datasets

In this study, we use data from two cohorts of RA patients: one in Austria and the other in Germany. We use the Austrian cohort for model training and validation and the German cohort to test on unseen data. Including these two datasets allows us to test model generalizability across different healthcare systems and treatment practices. This is necessary to ensure the robust model can be applied to diverse populations.

The Austrian dataset is sourced from BioReg, the Austrian Registry for Biologicals, Biosimilars, and targeted synthetic DMARDs^23^. This nationwide registry was established in 2010 to track the safety and efficacy of these treatments. BioReg includes patients diagnosed with RA, psoriatic arthritis, and spondyloarthritis treated in private offices and outpatient clinics in academic as well as non-academic settings nationwide under the ethical approval GS4-EK-4/085-2009. For this study, we included data from RA patients treated with bDMARDs between 2010 and 2024. Baseline clinical data were collected at the initiation of treatment, and remission was assessed at the 6-month follow-up. The Austrian dataset consists of data from 1052 patients.

The German dataset consists of anonymized clinical and laboratory data from RA patients at the University Hospital of Erlangen, Germany^13^. This dataset was part of a prospective observational cohort study, collected under ethical approvals 334-18 B and 333-16 B. Similar to the BioReg dataset, baseline data were collected at initiation and remission data at the 6-month follow-up. The German dataset consists of data from 154 patients.

The clinical characteristics were gathered following the same healthcare protocols and guidelines throughout the study period. Demographic information such as age and gender was recorded, as well as disease-specific characteristics like the medications patients were taking alongside bDMARDs, including csDMARDs. Disease-specific characteristics also included Disease Activity Score-28 (DAS 28), C-reactive protein (CRP) level, erythrocyte sedimentation rate (ESR), and rheumatoid factor (RF). Additionally, disease activity measures were assessed, including tender joint count (TJC28), swollen joint count (SJC28), and health assessment questionnaire (HAQ). The complete list of features can be seen in Tables 1 and 2.

### Data preprocessing

During preprocessing, all features, such as gender and visit dates, were converted into a numerical format. We sorted the visits of patients based on their time of visits and calculated the time between each visit and the patient’s first visit. Patients without a 6-month follow-up were excluded, as this was a necessary measure to assess remission at that time point. Additionally, patients missing DAS28-ESR scores at the 6-month follow-up were also removed from the dataset. Missing data for baseline clinical variables like DAS28-ESR scores were recalculated using available components like TJC28, CRP, and ESR, in accordance with standard clinical formulas^24^.

The proportion of remaining missing data for each feature is detailed in Supplementary Table 1 for the Austrian dataset and Supplementary Table 2 for the German dataset. We employed the Multiple Imputation by Chained Equations (MICE) method to handle missing values in our dataset. MICE is a well-established approach capable of imputing both continuous and categorical variables and is particularly suitable when data are assumed to be missing at random (MAR)^25,26^. Unlike univariate imputation techniques, MICE models each incomplete variable conditionally on the others, offering greater flexibility and robustness in complex clinical datasets. Although traditionally applied in settings with low to moderate missingness, recent simulation studies and applied research have shown that MICE can yield valid and unbiased results even when the proportion of missing data reaches 40–50%^27–29^. This approach enabled us to preserve the full sample size and reduce the risk of bias associated with complete-case analysis.

We ensured no data leakage or bias between the two datasets, and the outcome data was not used to impute missing values. The resulting datasets were refined to 1052 patient data for the Austrian dataset and 154 patient data for the German dataset.

Feature names and formats were standardized across both datasets to maintain compatibility between the two datasets; see Supplementary Tables 1 and 2. This included using consistent naming conventions and adjusting scales where necessary to align features. We selected features that are routinely collected, consistently defined, and commonly available across standard rheumatology workflows to ensure model robustness and reproducibility. These widely used clinical measures are reliably documented in diverse healthcare settings. Variables with inconsistent availability across both datasets (e.g., comorbidities, anti-cyclic citrullinated peptide antibody (anti-CCP) status) were excluded to prevent bias and support generalizable model development.

### Study goals, remission criteria, and validation strategy

This study has two primary aims. The first aim is to predict remission six months after starting the bDMARDs treatment, using baseline clinical data available at treatment initiation. Remission is defined as a DAS28-ESR score below 2.6 at the six-month follow-up, according to EULAR guidelines^4,30^.

The second aim is to develop a risk stratification system for patients based on their likelihood of achieving remission within six months.

In this study, we stratified patients based on their calibrated predicted probability of remission. Accurate probability estimates allow us to categorize patients into the following risk categories:

- *Low risk*: Patients with a predicted probability of remission greater than 66% were classified as low risk.
- *Moderate risk*: Patients with predicted probabilities between 33% and 66% were categorized as moderate risk.
- *High risk*: Patients with a predicted probability of remission below 33% were classified as high risk.

The intention was to move past binary classification into risk stratification to support clinical prioritization. Although the specific thresholds chosen here would be expected to change in different deployments, they illustrate our approach, whereby high-risk patients should be prioritized for closer monitoring and more aggressive interventions. In contrast, moderate-risk patients would require frequent but less urgent follow-ups. Patients classified as low-risk could be monitored less frequently. Clinicians may adjust the threshold for risk categories based on their institutional protocols or patient population or reflect a more cautious approach to follow-up. This approach would help optimize clinical resources while ensuring appropriate care for each risk group.

The Austrian Bioreg dataset was used for model training and validation, while the German Erlangen dataset was reserved for external validation as an unseen test set to ensure model robustness across different populations.

### Models

#### Remission prediction

Following literature^13,21^, we applied four machine learning algorithms-AdaBoost, XGBoost, Support Vector Machine (SVM), and Random Forest- to predict remission. Each model followed a standardized pipeline incorporating hyperparameter tuning and model calibration to ensure robust performance.

The Bioreg dataset was used for training, internal validation, and model calibration. Initially, 20% of the dataset was reserved as the calibration set, and the remaining data underwent five-fold cross-validation. During each iteration, four folds were used for training, while one fold served as the validation set. This process was repeated five times, ensuring each fold was used once for validation. Hyperparameters were tuned within this cross-validation process.

Within each fold, grid search was employed for hyperparameter optimization using the scikit-learn library function GridSearchCV. We selected the best configuration for each model based on cross-validated accuracy.

#### Calibration of predictive models for risk stratification

Following the cross-validation process, we selected the best-performing models optimized for features and hyperparameters. We calibrated the models using the reserved calibration set before testing them on the external dataset.

Calibration is essential to ensure that the predicted probabilities accurately reflect the likelihood of remission^31^. Indeed, it enables us to ensure that probabilities predicted by the models are accurate and reliable. To this end, we calculated calibration curves and applied four calibration techniques:

- Platt scaling^32^: A logistic regression model is applied to the classifier’s output probabilities, adjusting them to align with observed outcomes. Platt scaling assumes a parametric form, making it effective when probabilities follow a structured distribution.
- Isotonic regression^33^: A non-parametric method that fits a piecewise constant function, offering flexibility for cases where the relationship between predicted and actual outcomes is complex or non-linear.
- Beta calibration^34^: A parametric technique based on the Beta distribution, which adjusts probabilities by fitting two parameters.
- Spline calibration^35^: A regression model using spline functions to smooth the relationship between predicted probabilities and actual outcomes, improving the fit for non-linear patterns.

The choice to use multiple calibration techniques stems from recognizing that different methods may perform better depending on the model and data distribution. For example, Platt scaling works well with parametric relationships, while non-parametric methods like isotonic regression handle more complex relationships. We apply various techniques to assess the most reliable probability estimates for remission prediction.

After calibration, the predicted probabilities offer estimates of a patient’s likelihood of remission. For instance, a probability of 80% would suggest that historically, 80 out of 100 patients with similar profiles achieved remission. Calibration improves the dependability of probability estimates for clinical decision-making.

Accurate probability estimates provide clinicians with reliable assessments of patient risk, enabling informed decisions regarding treatment strategies and follow-up care. By incorporating this risk stratification approach, clinicians can allocate resources efficiently. They can focus on high-probability non-remission patients who need more attention while providing appropriate care to lower-risk patients without unnecessary interventions. This personalized approach could enhance patient outcomes and the overall efficiency of clinical management.

#### Model evaluation

After calibration, the models were evaluated on the Erlangen dataset test set. This previously unseen test set allowed for a robust evaluation of the models’ ability to generalize to new and unseen patient populations. Each model’s performance was assessed using the following classification metrics: precision, recall, F1-score, Matthews correlation coefficient (MCC), and area under the ROC curve (AUC-ROC). These metrics provide insights into the models’ effectiveness in deciding whether a patient is likely to achieve remission.

In addition to classification metrics, the Brier score^36^ was used to assess the reliability of the predicted probabilities. To that end, the Brier score measures how close the predicted probabilities are to the actual outcomes. A lower Brier score indicates better-calibrated models, meaning the predicted probabilities align more closely with the true remission probabilities.

The selection of the final model depends on the two primary clinical objectives: The first aim is to make an accurate binary decision about whether a patient will achieve remission at 6 months. We then prioritize classification metrics such as accuracy, F1 Score, and MCC. The second aim is to assess the likelihood of remission and to stratify patients into different risk categories. For this goal, we use calibration metrics such as the Brier score. Thus, the final model for clinical application should be chosen based on its ability to balance these two aspects. For decision-making tasks, the model that performed best in terms of classification metrics should be favored, while for tasks requiring accurate probability estimates and risk stratification, the model with the lowest Brier score should be preferred. Therefore, the choice of the final model depends on the practitioners’ specific clinical objectives.

#### Explainability of models

To better understand model predictions, we used SHapley Additive exPlanations (SHAP) to analyze feature importance and quantify the contribution of individual baseline features to the model’s predictions^37^. SHAP values were calculated for each model, indicating both the magnitude and direction of each baseline feature’s impact on the outcome.

SHAP determines each baseline feature’s contribution by comparing the model’s output with and without that feature. This process helps clinicians understand how individual variables influence the likelihood of remission in patients undergoing bDMARD therapy. We generated SHAP summary and feature importance plots, allowing clinicians to identify which features have the most significant impact easily. These plots support the development of personalized treatment strategies.

**Fig. 1:**
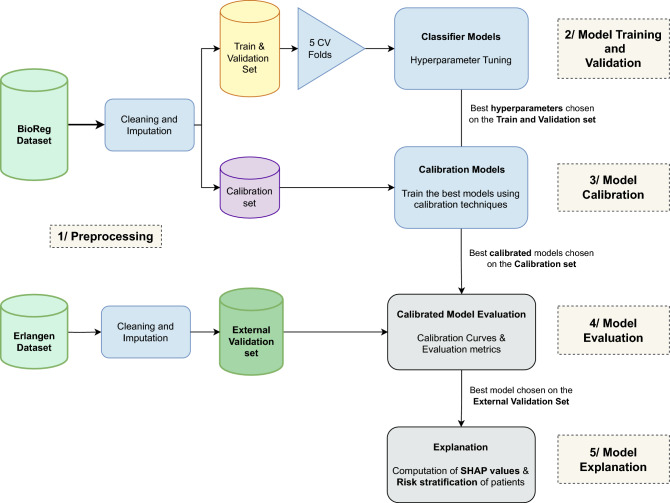
Flowchart illustrating the comprehensive workflow for predicting remission in RA patients. The process includes data preprocessing, model training, calibration, and evaluation. The BioReg dataset was used for training, validation, and calibration. To optimize performance, machine learning models, including AdaBoost, XGBoost, Support Vector Machine, and Random Forest, were trained using five-fold cross-validation and hyperparameter tuning via GridSearchCV. After model development, calibration techniques (Platt scaling, Isotonic regression, Beta calibration, and Spline calibration) were applied to improve the reliability of predicted probabilities. The models were evaluated on the external Erlangen dataset to assess generalizability, with key metrics including Brier score, accuracy, precision, recall, F1-Score, MCC, and ROC-AUC. Additionally, SHAP plots were generated to interpret the models’ predictions and identify the most influential features. The final step involved patient risk stratification based on predicted remission probabilities, supporting clinicians in personalized treatment planning.

The Flowchart in Fig. 1 details the model training and validation on the German and Austrian datasets.

### Software

We conducted all data preprocessing, model training, evaluation, and analysis using Python 3.9, with libraries such as Scikit-learn, Pandas, NumPy, betacal, and XGBoost.

## Results

### Patient demographics and baseline characteristics

In this study, we initially included 4344 patients from the Bioreg dataset. After applying inclusion criteria and removing patients who had not had any follow-up visits, 1,494 patients remained. Next, we excluded patients who did not have the DAS28-ESR Score recorded at the six-month follow-up. This resulted in removing 271 patients, leaving a final cohort of 1,223 patients. Further data cleaning was performed to remove visits that were not necessary for the predictive modeling (see “Data preprocessing” section). After this process, patients were labeled based on their remission status at six months according to the DAS28-ESR score. Table 1 presents the baseline characteristics of the 1,223 patients, including the mean and standard deviation (SD) or percentage of key clinical features. The table also compares baseline characteristics between patients who achieved remission and those who did not at six months. Of the 183 RA patients screened at Erlangen, 154 had at least one follow-up visit six months from baseline. The remaining 29 patients were excluded for not meeting this criterion. Table 2 summarizes the baseline clinical characteristics of the 154 RA patients, stratified by their six-month response.

**Table 1:** Mean (± standard deviation) and population percentage for each variable at baseline for RA patients treated with bDMARDs in the Bioreg dataset.

Characteristic	Overall (n = 1052)	Remission (n = 520)	Non-remission (n=532)
Age (years)	58.04 (12.95)	55.51 (13.34)	60.52 (12.04)
Gender (female %)	77.0	77.5	78.0
Swollen 28-joint count (SJC28)	2.49 (2.68)	1.79 (2.53)	3.17 (2.65)
Tender 28-joint count (TJC28)	3.57 (4.71)	2.07 (3.35)	5.03 (5.34)
Disease activity score 28 (DAS28)	3.31 (1.48)	2.60 (1.34)	4.00 (1.26)
Clinical disease activity index (CDAI)	11.87 (9.78)	8.58 (8.72)	15.09 (9.69)
Visual analogue scale (VAS) (physician assessment)	26.03 (19.09)	20.12 (17.37)	31.79 (18.94)
Visual analogue scale (VAS) (patient assessment)	37.43 (23.37)	29.99 (21.66)	44.70 (22.69)
Health assessment questionnaire (HAQ) Score	0.96 (0.64)	0.75 (0.56)	1.17 (0.65)
Erythrocyte sedimentation rate (ESR) (mm)	19.45 (17.06)	14.34 (14.04)	24.45 (18.23)
C-reactive protein (CRP) (mg/l)	8.53 (14.47)	6.75 (12.90)	10.27 (15.67)
Rheumatoid factor (RF) (positive%)	65.11	65.38	64.84
Weight (kg)	74.55 (15.40)	73.76 (15.24)	75.33 (15.51)
Conventional synthetic DMARDs (%csDMARDs)	65	68	63

**Table 2:** Mean (± standard deviation) and population percentage for each variable at baseline for RA patients treated with bDMARDs in the Erlangen dataset.

Characteristic	Overall (n=154)	Remission (n=77)	Non-remission (n=77)
Age (years)	53.47 (13.28)	50.97 (14.13)	55.96 (11.86)
Gender (female %)	73	66	79
Swollen 28-joint count (SJC28)	2.31 (4.12)	1.49 (3.44)	3.14 (4.55)
Tender 28-joint count (TJC28)	3.36 (5.08)	1.37 (2.90)	5.35 (5.93)
DAS28-ESR Score	3.10 (1.45)	2.24 (1.07)	3.97 (1.24)
CDAI Score	9.36 (10.25)	4.45 (5.87)	14.26 (11.30)
Visual analogue scale (VAS) (patient)	32.34 (25.46)	17.97 (18.50)	46.70 (23.26)
Visual analogue scale (VAS) (physician)	19.17 (19.80)	8.98 (10.50)	29.35 (21.60)
Health assessment questionnaire (HAQ)	0.90 (0.76)	0.59 (0.65)	1.22 (0.74)
Erythrocyte sedimentation rate (ESR) (mm)	17.21 (16.47)	11.49 (9.32)	22.92 (19.77)
C-reactive protein (CRP) (mg/l)	3.50 (4.80)	2.91 (4.53)	4.22 (4.89)
Rheumatoid factor (RF) (positive%)	97.4	94.8	100
Weight (kg)	77.10 (18.30)	75.23 (14.61)	78.62 (20.67)
Conventional synthetic DMARDs (% csDMARDs)	67	69	65

### Predictive models

#### Classification performance before calibration

The predictive models AdaBoost, Random Forest, Support Vector Machine (SVM), and XGBoost, were trained and evaluated on the Bioreg dataset. After hyperparameter tuning, their performance was assessed on an external test dataset (Erlangen), representing an independent patient population. The evaluation metrics, summarized in Table 3, reflect the ability of each model to predict remission after 6 months and generalize to unseen data.

Among the models, AdaBoost demonstrated the most consistent performance across the majority of metrics, showcasing its ability to strike a balance between sensitivity (recall) and precision. XGBoost, on the other hand, achieved the highest AUC-ROC, indicating superior discrimination capability in distinguishing between remission and non-remission cases. Despite this, AdaBoost emerged as the most balanced model overall, particularly for predicting remission after six months, due to its strong performance across multiple metrics and its ability to generalize effectively to the external test dataset. The results highlight the strengths of ensemble methods like AdaBoost and XGBoost, which combine predictions from multiple learners to improve accuracy and robustness. Ensemble models are generally more resilient to overfitting and better equipped to handle the variability inherent in external datasets, as demonstrated by their performance on the Erlangen dataset.

For a visual comparison of the models’ performance, refer to Supplementary Fig. 1.

**Table 3:** Model performance on external test dataset (Erlangen).

Model	Accuracy	Precision	Recall	F1-score	MCC	AUC-ROC
AdaBoost	85.71	0.88	0.83	0.85	0.72	0.87
Random forest	84.42	0.85	0.83	0.84	0.68	0.88
SVM	75.97	0.73	0.82	0.77	0.52	0.84
XGBoost	83.77	0.85	0.82	0.83	0.67	0.89

#### Model calibration and performance

**Fig. 2:**
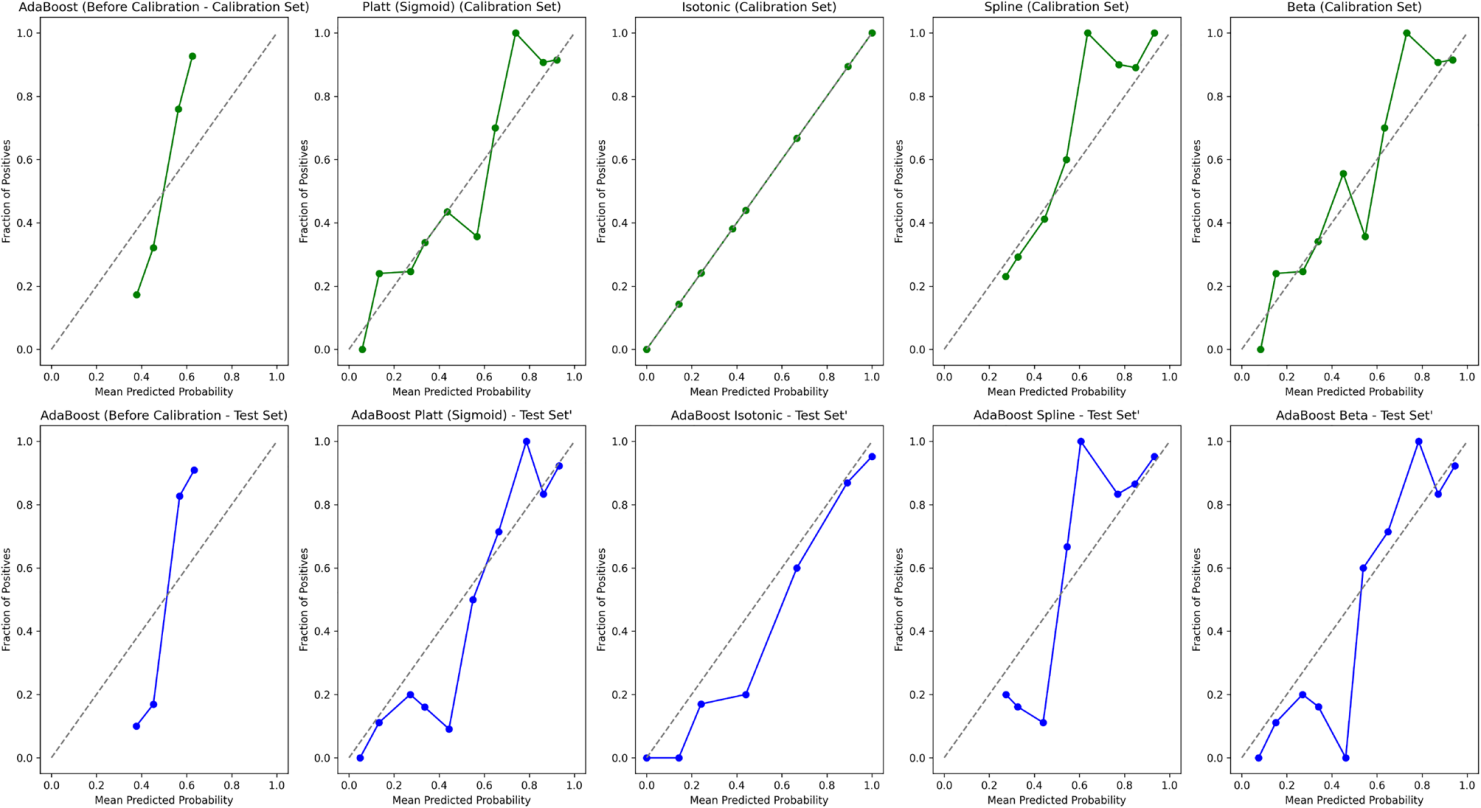
Calibration curves for AdaBoost across different calibration techniques (Platt, Isotonic, Spline, and Beta) for the calibration and test datasets. The isotonic calibration method provided the best alignment between predicted and observed probabilities, on both the calibration set and the test set.

**Fig. 3:**
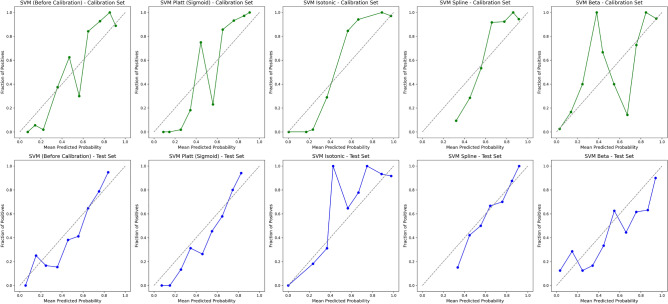
Calibration curves for SVM before and after different calibration techniques (Platt, Isotonic, Spline, and Beta) for the calibration and test availsets.

**Fig. 4:**
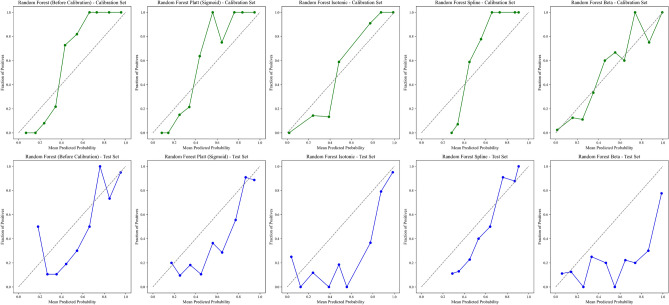
Calibration curves for Random Forest before and after different calibration techniques (Platt, Isotonic, Spline, and Beta) for the calibration and test availsets.

**Fig. 5:**
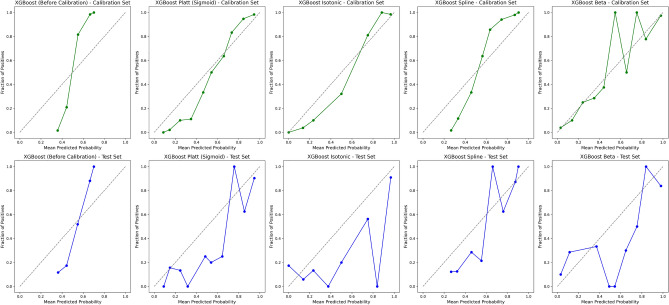
Calibration curves for XGBoost before and after calibration.

We evaluated the calibration performance of four models to assess the reliability of their probability estimates in predicting remission. The calibration curves assess the alignment between the predicted probabilities and the observed outcomes. We applied various calibration methods to refine these estimates, including Platt scaling (sigmoid), Isotonic regression, Spline calibration, and Beta calibration. Figures 2, 3, 4, and 5 show the calibration curves for each model before and after calibration. Model performances were evaluated using Brier score, Accuracy, Precision, Recall, F1-Score, and Matthews Correlation Coefficient (MCC). Tables 4, 5, 6, and 7 summarize the results before and after calibration.

#### AdaBoost calibration performance

The calibration curves for the AdaBoost model (Fig. 2) illustrate the impact of calibration techniques on the reliability of the predicted probabilities. The diagonal line, shown as a gray reference in the calibration plot, represents perfect calibration, where the predicted probabilities align exactly with the observed outcomes. In other words, a predicted probability of 0.7 would correspond to an actual event rate of 0.7, and so on. Before calibration, the predicted probabilities of the uncalibrated AdaBoost model showed deviations from the ideal diagonal line, suggesting some degree of over- and underestimation in the predictions. This is further supported by the higher pre-calibration Brier score of 0.20, indicating room for improvement in the predicted probabilities.

After applying calibration techniques, isotonic regression achieved the best overall calibration performance for the calibrated AdaBoost model, reducing the Brier score to 0.13 and closely aligning the predicted probabilities with the observed outcomes. Spline and beta calibrations also improved, with Brier scores of 0.14, while maintaining high classification performance metrics.

The summary of performance metrics for both the uncalibrated AdaBoost model and the calibrated AdaBoost models is presented in Table 4.

**Table 4:** Performance metrics for AdaBoost before and after different calibration techniques (Platt, Isotonic, Spline, and Beta) for the calibration and test datasets.

Method	Brier	Acc. (%)	Prec.	Rec.	F1	MCC	AUC
No calibration	0.20	85.71	0.88	0.83	0.85	0.72	0.87
Sigmoid	0.14	84.42	0.84	0.84	0.84	0.69	0.87
Isotonic	0.13	85.06	0.88	0.82	0.85	0.70	0.87
Spline	0.14	85.71	0.88	0.83	0.85	0.72	0.86
Beta	0.14	85.06	0.86	0.84	0.85	0.70	0.87

#### SVM calibration performance

The calibration curves for the SVM model (Fig. 3) show deviations from the ideal diagonal line in the uncalibrated model, particularly in the lower- and middle-range of the predicted probabilities. This indicates that the uncalibrated SVM model overestimated probabilities, assigning higher likelihoods to positive outcomes than warranted. The pre-calibration Brier score of 0.17 further highlights the need for refinement in probability estimation.

After calibration, Beta and Spline methods offered slight improvements, particularly in aligning predicted probabilities with observed outcomes in the mid-range probabilities. However, the calibrated SVM models still exhibited miscalibration at the extremes of the probability distribution, and the Brier score marginally increased to 0.18 after Beta calibration, suggesting limited success in improving overall probability estimates.

From a classification perspective, minor improvements were observed following calibration. The F1-Score increased from 0.77 to 0.78, and the Matthews Correlation Coefficient (MCC) improved from 0.52 to 0.54, indicating slightly better agreement between predictions and actual outcomes. Recall rose modestly from 0.82 to 0.84, while precision remained stable. These changes suggest that calibration had a limited but measurable impact on the model’s ability to classify remission outcomes correctly.

While calibration techniques brought some predicted probabilities closer to the ideal diagonal line, particularly in mid-range probabilities, they did not resolve miscalibration at the extremes. The results suggest that the calibrated SVM models, while showing modest improvements in classification metrics, cannot be reliably used for clinical applications. The performance metrics for the SVM model before and after calibration are summarized in Table 5.

**Table 5:** Performance metrics for SVM before and after different calibration techniques (Platt, Isotonic, Spline, and Beta) for the calibration and test availsets.

Method	Brier	Acc. (%)	Prec.	Rec.	F1	MCC	AUC-ROC
No calibration	0.17	75.97	0.73	0.82	0.77	0.52	0.84
Sigmoid	0.18	74.03	0.70	0.84	0.76	0.49	0.84
Isotonic	0.17	75.32	0.77	0.71	0.74	0.51	0.82
Spline	0.18	75.97	0.74	0.79	0.77	0.52	0.83
Beta	0.18	76.62	0.73	0.84	0.78	0.54	0.84

#### Random forest calibration performance

The Random Forest model exhibited a relatively well-calibrated pre-calibration curve compared to other models (Fig. 4). The pre-calibration curve shows that the predicted probabilities were already reasonably well-aligned with the actual outcomes, as indicated by a Brier score of 0.157 and a high classification accuracy of 84.42%. Additionally, the model achieved an F1-Score of 0.842 and a Matthews Correlation Coefficient (MCC) of 0.689, reflecting strong classification performance before calibration.

Various calibration methods yielded mixed results. Spline calibration produced the best calibration results, with the post-calibration curves demonstrating better alignment with the diagonal, particularly in the middle probability range. However, Beta calibration worsened the probability estimates, leading to an increase in the Brier score to 0.226. This increase in the Brier score indicates that Beta calibration did not improve the calibration quality.

Despite the increase in the Brier score, recall improved from 0.831 to 0.909 with Beta calibration, showing better sensitivity in identifying true positives. However, this came at the expense of accuracy, which dropped to 75.32%, and the F1-Score also declined to 0.787. After calibration, the MCC dropped to 0.533, suggesting a less balanced overall classification performance.

In contrast, Spline calibration resulted in more consistent performance, with the post-calibration curve closely aligning with the diagonal. While the overall classification performance did not significantly improve, the calibrated probabilities showed better accuracy across a broader range of predicted probabilities, especially in the middle ranges, without introducing significant degradation to the Brier score.

In summary, Spline calibration provided better alignment with the diagonal line and more stable performance, while Beta calibration led to a deterioration in calibration quality. Therefore, Spline calibration can be considered the more effective method for improving the calibration of the Random Forest model.

**Table 6:** Performance metrics for Random Forest before and after different calibration techniques (Platt, Isotonic, Spline, and Beta) for the calibration and test availsets.

Method	Brier	Acc. (%)	Prec.	Rec.	F1	MCC	AUC-ROC
No calibration	0.16	84.42	0.85	0.83	0.84	0.69	0.88
Sigmoid	0.17	75.97	0.70	0.91	0.79	0.54	0.88
Isotonic	0.19	76.62	0.71	0.91	0.80	0.56	0.88
Spline	0.15	81.17	0.77	0.90	0.83	0.63	0.88
Beta	0.23	75.32	0.69	0.91	0.79	0.53	0.88

#### XGBoost calibration performance

Figure 5 presents the calibration curves for XGBoost on both internal and external validation availablesets. Without calibration, the probability estimates could be unreliable. The model overestimated probabilities in the lower ranges and underestimated them in the higher ranges, as indicated by a Brier score of 0.179. Despite this, the uncalibrated model performed well, achieving an accuracy of 83.77%, an F1 score of 0.834, and a Matthews Correlation Coefficient (MCC) of 0.676.

Substantial improvements in probability estimates were observed after calibration, particularly with beta calibration. Beta calibration reduced the Brier score from 0.179 to 0.152. Furthermore, while the accuracy decreased slightly to 80.52%, the recall increased from 0.818 to 0.883, indicating a better ability to identify true positives. The F1 score remained relatively high at 0.819, and the MCC was 0.618 after calibration. The AUC-ROC score remained stable at 0.889, demonstrating consistent discriminative ability before and after calibration.

Overall, Beta calibration provided the best calibration performance for the XGBoost model, leading to more accurate probability estimates, as reflected by the lower Brier score. While there was a slight trade-off in accuracy, the improvements in recall and calibrated probability estimates make Beta calibration the preferred method for XGBoost.

**Table 7:** Performance metrics for XGBoost before and after different calibration techniques (Platt, Isotonic, Spline, and Beta) for the calibration and test availablesets.

Method	Brier	Acc. (%)	Prec.	Rec.	F1	MCC	AUC-ROC
No calibration	0.18	83.77	0.85	0.82	0.83	0.68	0.89
Sigmoid	0.15	79.22	0.74	0.90	0.81	0.60	0.89
Isotonic	0.15	81.17	0.77	0.88	0.82	0.63	0.88
Spline	0.15	79.87	0.76	0.88	0.81	0.61	0.89
Beta	0.15	80.52	0.76	0.88	0.82	0.62	0.89

### Best model for remission prediction and probability estimates

AdaBoost provided the best performance for remission classification and probability accuracy among all models tested without calibration. It achieved the highest accuracy (86.36%), F1-Score (0.86), and MCC (0.73), demonstrating strong classification capabilities. Additionally, its Brier Score of 0.134 indicated that it produced the most accurate probability estimates, outperforming the other models.

Calibration methods such as Spline and Beta improved the probability estimates of models like Random Forest and XGBoost, but they did not surpass AdaBoost’s performance.

In summary, AdaBoost with isotonic regression was the most effective model overall, making it the best choice for both accurate remission classification and reliable probability estimates.

### Explainability and feature importance

**Fig. 6:**
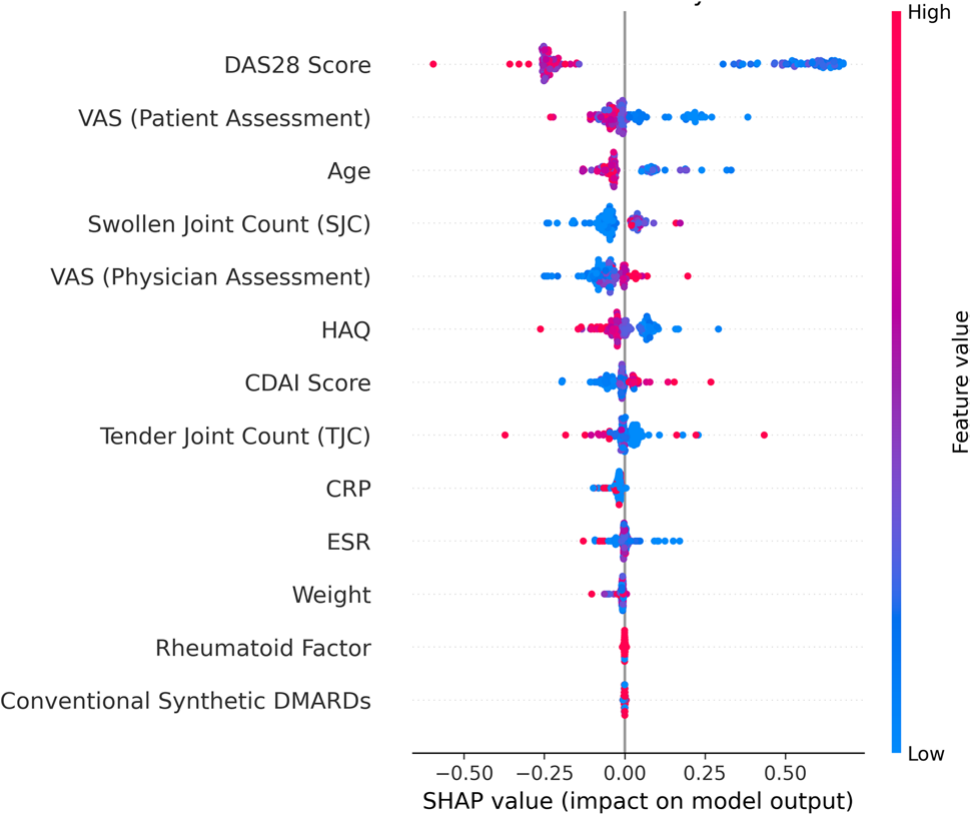
SHAP summary plot on the test availableset (Erlangen availableset) highlighting feature importance and directional impact on remission prediction. This plot illustrates the impact of baseline features on the model’s output for predicting six-month remission in rheumatoid arthritis patients before initiating bDMARD therapy. Each dot represents a patient, with the color indicating the baseline feature value (red for high, blue for low). Features like DAS28 Score, VAS (Patient Assessment), and Age have the highest influence, where higher DAS28 and VAS values at baseline reduce the likelihood of remission, while lower values increase it. This explainability supports clinicians in understanding how baseline clinical indicators influence predictions and informs personalized treatment decisions.

To enhance interpretability and clinical utility, SHapley Additive exPlanations (SHAP) were applied to the test availableset of the AdaBoost classifier to quantify the contribution of individual features to the model’s predictions. The SHAP summary plot in Fig. 6 illustrates the top baseline features that had the most significant influence on the model’s prediction of remission.

The plot shows that the DAS28 Score at the baseline was the most influential feature, indicating its strong predictive power for remission outcomes. Other important features include the VAS score (based on patient assessment), age, and SJC, which were critical in shaping the model’s predictions. The SHAP values highlight the relationship between feature values and their impact on the model. Higher values of features such as DAS28 and age shifted the predictions towards non-remission. In comparison, lower values of these features were associated with a higher likelihood of remission. This explainability offers valuable insights for clinicians by identifying the key factors contributing to remission prediction and clarifying their directional impact on outcomes, enabling more personalized and informed treatment decisions based on these critical clinical indicators.

### Risk stratification outcomes

The AdaBoost model demonstrated strong performance in estimating remission probabilities, with isotonic regression identified as the optimal calibration method. This was supported by a lower Brier score (0.13) and superior alignment between predicted and observed probabilities in calibration curves (Fig. 2).

**Fig. 7:**
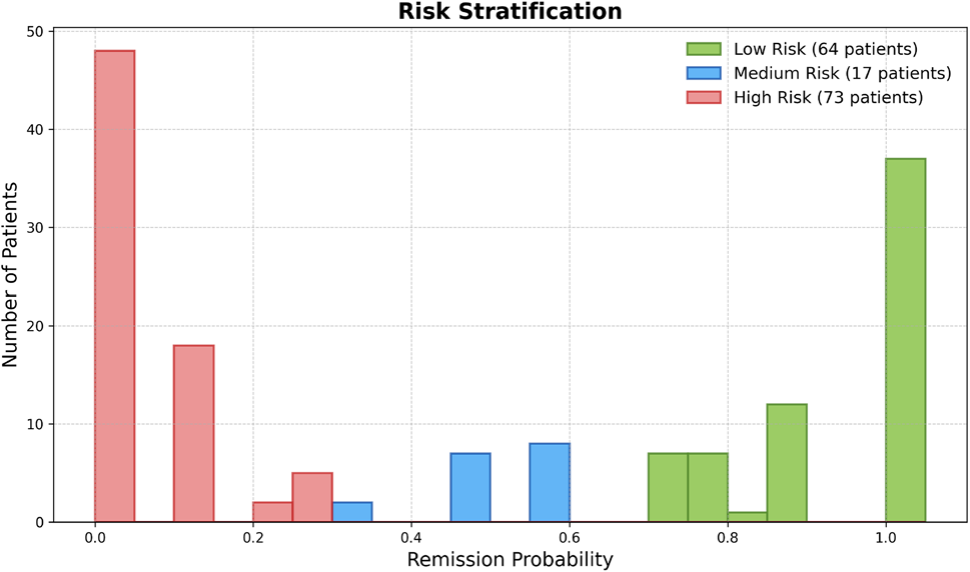
Risk stratification using the AdaBoost model calibrated with isotonic regression. Patients were categorized into low-risk (green), medium-risk (blue), and high-risk (red) groups based on their calibrated remission probabilities.

Following calibration, patients were stratified into three risk categories based on their predicted remission probabilities: low risk (>0.66), medium risk (0.33–0.66), and high risk (<0.33). These thresholds were chosen to reflect clinically meaningful separation in treatment response probabilities. Figure 7 illustrates the distribution of remission probabilities within each risk group in the test set.

Baseline characteristics of patients differed substantially across the risk groups (Table 8). Patients classified as high risk presented with more severe disease activity at baseline, including higher DAS28 and CDAI scores, elevated inflammatory markers (ESR and CRP), and greater joint involvement. In contrast, patients in the low-risk group exhibited milder clinical profiles.

Observed remission outcomes reflected the predicted risk levels (Table 9). The remission rate was 89.7% in the low-risk group, compared to 24.1% and 15.8% in the medium- and high-risk groups, respectively. This clear gradient in treatment response validates the model’s ability to stratify patients into clinically meaningful categories and supports its utility for precision medicine in RA.

**Table 8:** Mean (± standard deviation) and population percentage for each baseline variable across predicted remission risk groups (Low, Medium, High) for RA patients treated with bDMARDs in the Erlangen dataset.

Feature	Low risk (n=64)	Medium risk (n=17)	High risk (n=73)
Age (years)	49.8 (14.5)	49.3 (11.4)	59.9 (10.0)
Gender (female %)	73	66	79
Swollen 28-joint count (SJC28)	1.0 (3.2)	2.8 (3.7)	3.6 (4.9)
Tender 28-joint count (TJC28)	0.7 (1.4)	3.6 (4.0)	6.4 (6.5)
DAS28-ESR score	1.8 (0.5)	3.7 (0.9)	4.3 (1.2)
CDAI score	3.4 (3.7)	10.3 (8.6)	16.0 (12.1)
Visual analogue scale (VAS) (patient)	13.7 (13.9)	38.2 (18.0)	51.5 (24.0)
Visual analogue scale (VAS) (physician)	7.9 (8.7)	22.8 (17.1)	30.8 (23.2)
Health assessment questionnaire (HAQ)	0.5 (0.5)	0.6 (0.8)	1.5 (0.6)
Erythrocyte sedimentation rate (ESR) (mm)	9.3 (7.4)	18.6 (12.3)	25.9 (21.2)
C-reactive protein (CRP) (mg/l)	0.3 (0.5)	0.4 (0.5)	0.4 (0.5)
Rheumatoid factor (RF) (Positive%)	100	100	100
Weight (kg)	28.5 (20.5)	27.2 (6.3)	27.6 (5.2)
Conventional synthetic DMARDs (% csDMARDs)	70	80	60

**Table 9:** Observed remission rates within each predicted risk group in the Erlangen bDMARD-treated RA cohort.

Risk group	Remission rate (%)
Low risk (n = 64)	89.7%
Medium risk (n = 17)	24.1%
High risk (n = 73)	15.8%

## Discussion

This study highlights the potential of machine learning models, particularly ensemble learning methods, in predicting six-month remission and stratifying risk in RA patients starting bDMARDs therapy. By evaluating Random Forest, XGBoost, Support Vector Machines (SVM), and AdaBoost, alongside different calibration techniques, we established a robust framework for remission prediction and actionable risk stratification. One of the highlights of this study is the use of calibrated probabilities, providing reliable and clinically interpretable predictions.

Ensemble models, including XGBoost, AdaBoost, and Random Forest, outperformed the SVM model in this study. AdaBoost emerged as the best-performing model, achieving a classification accuracy of 85.71%, an F1-Score of 0.85, and the lowest Brier score of 0.13 after isotonic regression calibration. These metrics demonstrate its superior ability to align predicted probabilities with actual outcomes, making it well-suited for applications requiring reliable risk stratification. Random Forest and XGBoost also performed strongly, with accuracies of 84.42% and 83.77%, and Brier scores of 0.16 and 0.15, respectively, after calibration. Compared to AdaBoost, their slightly higher Brier scores suggest a need for further calibration refinement. In contrast, SVM struggled with probability calibration, achieving an accuracy of 75.97% and a Brier score of 0.17, reflecting its limitations in generating reliable probabilistic outputs.

The consistent performance of ensemble models highlights their suitability for this task. Their structures can capture non-linear relationships, and accommodate complex feature interactions-key characteristics of clinical availablesets. Among these, AdaBoost is the most reliable model due to its balance of accuracy, calibration, and interpretability, providing a valuable tool for guiding treatment decisions in RA patients.

Calibration played a crucial role in this study by improving the reliability of predicted probabilities and making them actionable for clinical decision-making. By comparing isotonic regression, beta calibration, and spline calibration, we demonstrated the importance of tailoring calibration techniques to specific models. The calibrated probabilities allowed for stratifying patients into low-, medium-, and high-risk groups, providing a nuanced approach to clinical prioritization. For example, high-risk patients can be prioritized for intensive monitoring, while low-risk individuals may require less frequent follow-ups, optimizing clinical resources.

The integration of SHapley Additive Explanations (SHAP) provided additional depth to our findings by quantifying the influence of clinical features on model predictions. Key predictors such as DAS28, HAQ score, age, and Swollen Joint Count (SJC) were identified as the most influential factors, with many results aligning well with established clinical knowledge, enhancing the interpretability of our models. For example, DAS28 and VAS (Patient Assessment) reflect known indicators of disease activity and patient health. However, some findings, such as the unexpected effect of SJC, where higher values sometimes correspond to remission, may reflect specific patterns in the availableset, such as aggressive treatment strategies or interactions captured by the model. While SHAP explanations are based on the available and may not always align with clinical intuition, they provide transparency by showing how features contribute to predictions. By bridging the gap between complex machine learning techniques and practical clinical applications, SHAP insights foster trust among clinicians, enabling more personalized treatment planning while encouraging further exploration of unexpected results^38^. Some of the patterns identified by the model may diverge from traditional clinical expectations, yet they could reveal novel relationships and generate new hypotheses that are worth further investigation in clinical research.

This study also overcomes several limitations of prior work. First, remission assessment was defined based on EULAR standards, and only routine clinical available was used, avoiding the need for costly or less accessible modalities like medical imaging or gene expression available. Second, we leveraged two availablesets from Austria and Germany, which differed in demographic and healthcare characteristics, to ensure the broader applicability of our findings. By evaluating model performance across these distinct cohorts, we demonstrated the ability of the models to generalize effectively. Many prior studies relied on small, homogeneous availablesets, limiting their relevance to broader populations. By contrast, our approach tested predictive models in diverse clinical environments, providing confidence in their real-world applicability. Third, calibration, often neglected in previous work, was rigorously evaluated here, providing reliable probability estimates essential for risk stratification. Finally, unlike studies focusing solely on binary outcomes, we showed the clinical value of stratifying patients into actionable risk categories, offering a framework for personalized treatment planning.

While this study addresses several key challenges in remission prediction and risk stratification in rheumatoid arthritis, there are limitations that future research could address. The study population was limited to Austria and Germany, which may not fully capture the variability across different healthcare systems, clinical practices, and patient lifestyles. Future research could validate the models using datasets from more diverse populations to enhance their robustness and generalizability. Additionally, this study focused on six-month remission as an endpoint, without evaluating sustained treatment responses at twelve months, which could provide further insights into long-term effectiveness. While SHAP analysis supported model interpretability, further exploration of the clinical implications of individual predictors is warranted to better align model outputs with medical reasoning and domain knowledge. Future studies may also explore the use of alternative remission definitions, such as the Simplified Disease Activity Index or Boolean criteria, where such data are available, to better reflect stricter and more specific clinical endpoints.

In this study, we selected routinely collected and consistently defined clinical features that were available across both cohorts. This allowed for harmonized analysis and ensured the development of models that reflect real-world rheumatology workflows. However, it is possible that incorporating a broader set of variables, including biomarkers, comorbidities, disease duration, and more detailed treatment histories, combined with advanced feature selection techniques, could further improve predictive performance. Future work using more comprehensive and prospectively harmonized datasets may enable the identification of additional clinically relevant predictors and support the development of even more accurate and generalizable models.

## Conclusion

This study applies machine learning methods to address the critical clinical challenge of predicting six-month remission and stratifying risk in RA patients initiating bDMARDs therapy. By leveraging routine baseline clinical data and established calibration techniques, we developed a reliable framework that supports personalized patient management.

The calibrated AdaBoost model outperformed other approaches, providing accurate remission probabilities and enabling effective stratification into clinically actionable risk groups. These insights facilitate tailored follow-up plans and optimize resource allocation. Furthermore, the incorporation of the explainable AI method (SHAP) ensures that predictions are interpretable and aligned with clinical relevance, which could empower healthcare professionals to make informed decisions.

This study employs established methods, their targeted application to bDMARDs outcomes in RA patients offers valuable insights into remission prediction. By validating the framework across diverse datasets and emphasizing interpretable outputs, we provide a practical tool that enhances precision medicine in RA management.

## Supplementary Information

Supplementary Information.

## Data Availability

The Erlangen dataset is available on Zenodo (https://doi.org/10.5281/zenodo.12507169), and the Bioreg dataset can be accessed upon request under the ethical approval guidelines of Bioreg. The code for this paper is available on GitHub (https://github.com/fatemehsalehi/PRECISE-RA).
