# Supplementary material for: A robust machine learning approach to predicting remission and stratifying risk in rheumatoid arthritis patients treated with bDMARDs
Source: Sci Rep. 2025 Jul 4;15:23960. doi: 10.1038/s41598-025-09975-z (PMC12227524; doi:10.1038/s41598-025-09975-z)
Supplement: Supplementary file 1 — Supplementary Information. [file 41598_2025_9975_MOESM1_ESM.pdf]

Appendix

Missing Data Details

Table 1. Percentage of Missing Data for Bioreg Clinical Features

Clinical Feature	Percentage Missing (%)
Age (years)	0.00
Gender	0.00
Swollen Joint Count-28 (SJC28)	19.76
Tender Joint Count-28 (TJC28)	12.34
Disease Activity Score in 28 Joints (DAS28-ESR)	1.76
Clinical Disease Activity Index (CDAI)	22.97
Visual Analog Scale (VAS) Activity (Patient)	17.03
Visual Analog Scale (VAS) Activity (Physician)	7.88
Health Assessment Questionnaire (HAQ) Score	27.64
Erythrocyte Sedimentation Rate (ESR)	9.03
C-Reactive Protein (CRP)	8.27
Rheumatoid Factor (RF)	0.00
Weight	1.02
Conventional Synthetic DMARDs (csDMARDs)	1.08

Table 2. Percentage of Missing Data for Erlangen Clinical Features

Clinical Feature	Percentage Missing (%)
Age (years)	0.00
Gender	0.00
Swollen Joint Count-28 (SJC28)	1.76
Tender Joint Count-28 (TJC28)	1.76
Disease Activity Score in 28 Joints (DAS28-ESR)	1.76
Clinical Disease Activity Index (CDAI)	14.88
Visual Analog Scale (VAS) Activity (Patient)	1.76
Visual Analog Scale (VAS) Activity (Physician)	14.88
Health Assessment Questionnaire (HAQ) Score	1.77
Erythrocyte Sedimentation Rate (ESR)	1.76
C-Reactive Protein (CRP)	0.00
Rheumatoid Factor (RF)	0.00
Weight	0.00
Conventional Synthetic DMARDs (csDMARDs)	0.00

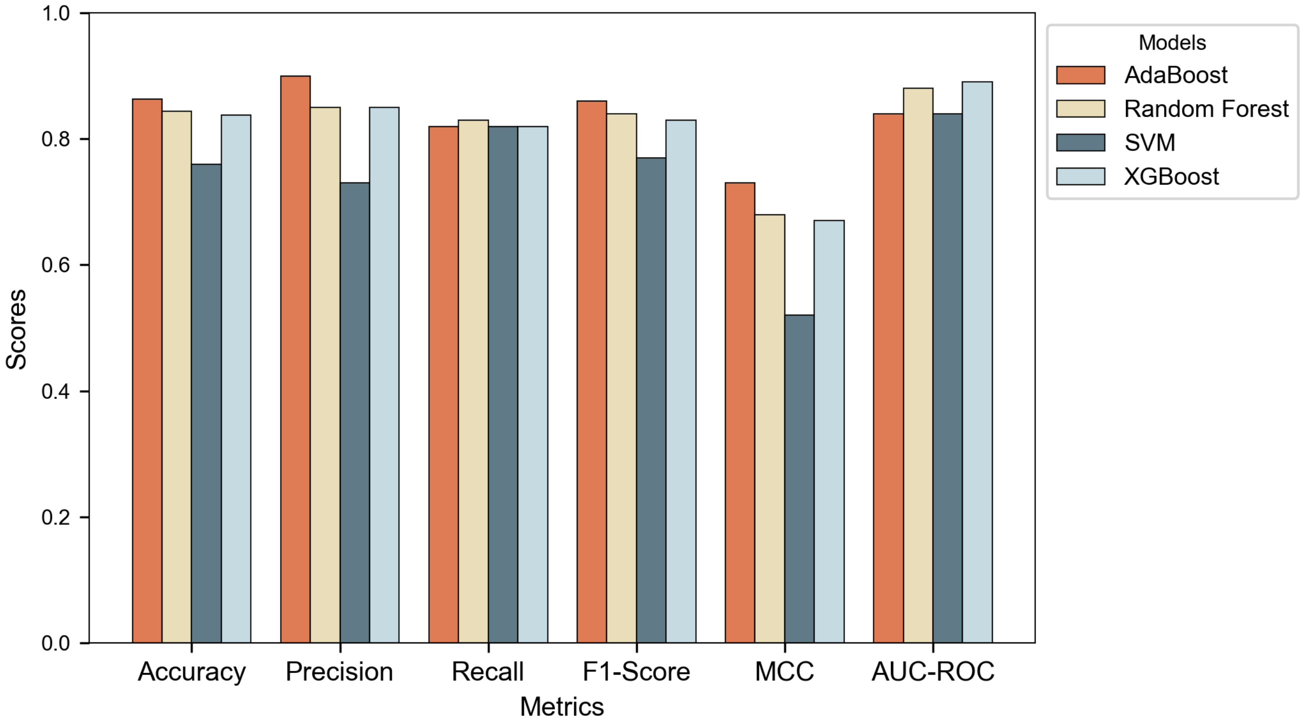

Figure 1. Validation metrics for external validation
